# Optimal Control of SonoVue Microbubbles to Estimate Hydrostatic Pressure

**DOI:** 10.1109/TUFFC.2019.2948759

**Published:** 2019-10-21

**Authors:** Amanda Q. X. Nio, Alessandro Faraci, Kirsten Christensen-Jeffries, Jason L. Raymond, Mark J. Monaghan, Daniel Fuster, Flemming Forsberg, Robert J. Eckersley, Pablo Lamata

**Affiliations:** 1 Department of Biomedical EngineeringSchool of Biomedical Engineering and Imaging SciencesKing’s College London 4616 LondonSE1 7EHU.K.; 2 Department of Engineering ScienceUniversity of Oxford 6396 OxfordOX1 3PJU.K.; 3 Department of CardiologyKing’s College Hospital 111990 LondonSE5 9RSU.K.; 4 Institut Jean Le Rond D’Alembert, Sorbonne Université 27063 Center National de la Recherche Scientifique, UMR 7190F-75005ParisFrance; 5 Department of RadiologyThomas Jefferson University 6559 PhiladelphiaPA19107USA

**Keywords:** Hydrostatic pressure, subharmonic imaging, ultrasound contrast agents

## Abstract

The measurement of cardiac and aortic pressures enables diagnostic insight into cardiac contractility and stiffness. However, these pressures are currently assessed invasively using pressure catheters. It may be possible to estimate these pressures less invasively by applying microbubble ultrasound contrast agents as pressure sensors. The aim of this study was to investigate the subharmonic response of the microbubble ultrasound contrast agent SonoVue (Bracco Spa, Milan, Italy) at physiological pressures using a static pressure phantom. A commercially available cell culture cassette with Luer connections was used as a static pressure chamber. SonoVue was added to the phantom, and radio frequency data were recorded on the ULtrasound Advanced Open Platform (ULA-OP). The mean subharmonic amplitude over a 40% bandwidth was extracted at 0–200-mmHg hydrostatic pressures, across 1.7–7.0-MHz transmit frequencies and 3.5%–100% maximum scanner acoustic output. The Rayleigh–Plesset equation for single-bubble oscillations and additional hysteresis experiments were used to provide insight into the mechanisms underlying the subharmonic pressure response of SonoVue. The subharmonic amplitude of SonoVue increased with hydrostatic pressure up to 50 mmHg across all transmit frequencies and decreased thereafter. A decreasing microbubble surface tension may drive the initial increase in the subharmonic amplitude of SonoVue with hydrostatic pressure, while shell buckling and microbubble destruction may contribute to the subsequent decrease above 125-mmHg pressure. In conclusion, a practical operating regime that may be applied to estimate cardiac and aortic blood pressures from the subharmonic signal of SonoVue has been identified.

## I. Introduction

Microbubble-based ultrasound contrast agents are currently used in the clinic to complement standard B-mode imaging across multiple organs and systems in the human body, including the heart, breast, and liver [1]–[4]. In the heart, the commercially available contrast agents SonoVue/Lumason (Bracco Spa, Milan, Italy), Luminity/Definity (Lantheus Medical Imaging Inc., North Billerica, MA, USA), and Optison (GE Healthcare, Princeton, NJ, USA) may be applied to assess left ventricular function, structural left ventricular abnormalities, and myocardial perfusion [2], [3]. In addition, these microbubbles have a promising new application as pressure sensors [5], [6], which would enable minimally invasive estimations of the cardiac and large artery pressures underpinning diagnostic information on cardiac contractility and stiffness [7]–[9]. Successful implementation of microbubble-based cardiac pressures would provide a safer and more cost-effective alternative to the current clinical method that requires invasive cardiac catheterization [8], [9].

Using single-element transducers, the ultrasound contrast agents Levovist, Optison, Definity, ZFX, and Sonazoid have been found to generate a subharmonic signal that is linearly and negatively correlated with static hydrostatic pressures from 0 to 186 mmHg [10]. This phenomenon may be due to the changes in bubble surface tension and shell buckling [11], [12] and is additionally affected by ultrasound settings, such as transmit frequency, pulse length, and acoustic pressure. As acoustic pressure increases, the subharmonic signal of ultrasound contrast agents can be delineated into three distinct phases: 1) occurrence; 2) growth; and 3) saturation (see Fig. 3) [13], [14]. In the occurrence and saturation phases, the subharmonic amplitude is stable despite an increase in acoustic pressure. In contrast, the growth phase is characterized by an increase in subharmonic amplitude with acoustic pressure. An acoustic pressure within the growth phase has been shown to be necessary to elicit a strong negative linear relationship between the subharmonic amplitude of ultrasound contrast agents and the hydrostatic pressure [6], [13].

**Fig. 1.**
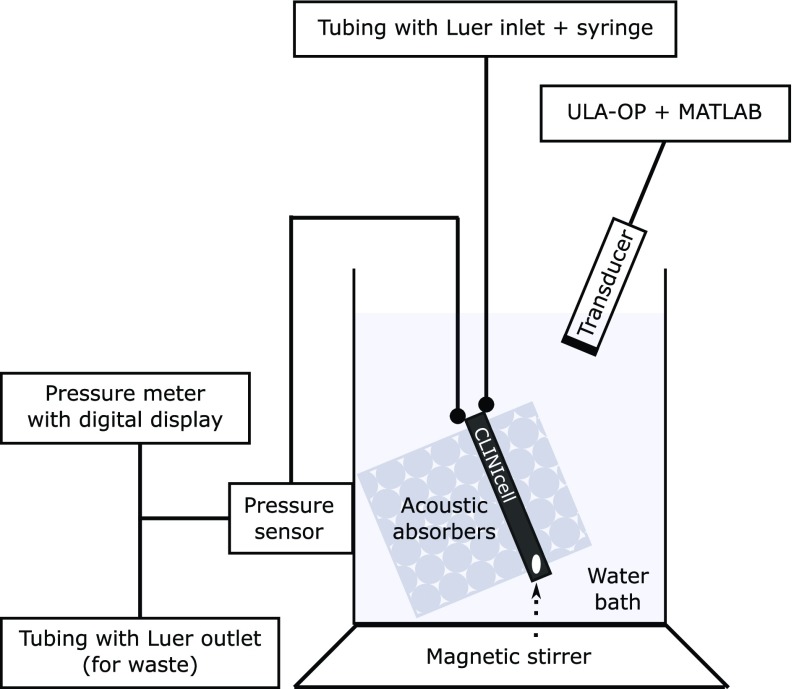
Schematic of the static pressure phantom. The CLINIcell was submerged in a water bath with layers of acoustically absorbent foam in front of and behind it. The transducer was positioned at a 45° angle relative to the window of the CLINIcell. SonoVue was added to the CLINIcell with a syringe, via a Luer stopcock.

**Fig. 2.**
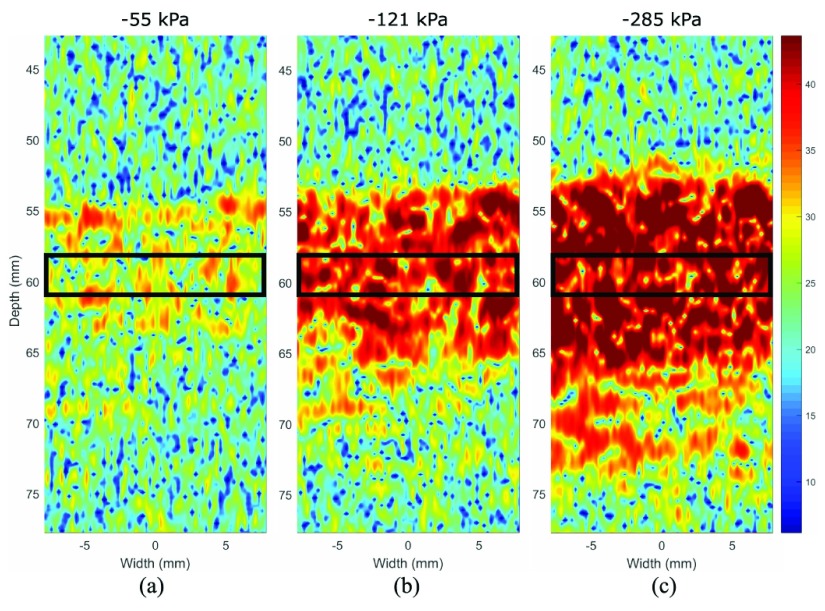
2-D images of the subharmonic signal at (a) 55-, (b) 121-, and (c) 285-kPa peak-negative acoustic pressures, illustrating a greater amplitude (dB) with increasing peak-negative acoustic pressure. In this example, hydrostatic pressure was maintained at 75 mmHg, and data were recorded at transmit frequency 4 MHz with 12-cycle pulses. The region of interest is demarcated by the black rectangular border.

**Fig. 3.**
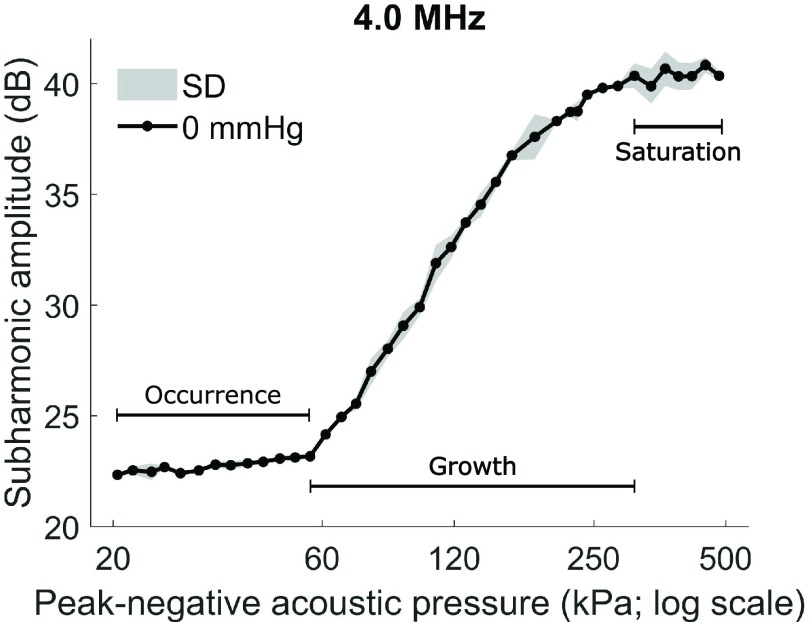
Mean subharmonic amplitude of SonoVue at ambient hydrostatic pressure (0 mmHg) across the full range of scanner acoustic pressures at a transmit frequency of 4 MHz (${n} \,\,={2}$). SD: 1 standard deviation.

Among the commercially available ultrasound contrast agents, Sonazoid has been investigated most extensively due to its greatest sensitivity to hydrostatic pressure following the study by Halldorsdottir *et al.* [10]. However, Sonazoid is currently not approved or marketed in Europe [15]. A potential alternative to Sonazoid is the ultrasound contrast agent SonoVue marketed by Bracco Spa, which is widely used in Europe [3]. To the best of our knowledge, there have been three investigations of SonoVue as a potential pressure sensor [16]–[18], and none of these have been done by the research group spearheading current efforts with Sonazoid. The first study was by Andersen and Jensen [16], who in fact examined the ratio of the subharmonic to fundamental components, instead of the subharmonic as described by Halldorsdottir *et al.* [10]. The second study by Sun *et al.* [18] found an increase in the subharmonic signal with hydrostatic pressure when excited by a 1.33-MHz ultrasound pulse at an acoustic pressure of 300 kPa, but a decrease when excited by a 4-MHz pulse at a similar acoustic pressure (300 kPa). In contrast, the third study by Li *et al.* (which was published in 2018 while data collection for this current study was ongoing) [17] reported a decrease in the subharmonic signal with hydrostatic pressure when excited by a 1.33-MHz ultrasound pulse at an acoustic pressure of 350 kPa, but an increase from 0- to 50-mmHg hydrostatic pressure at 4 MHz and the same acoustic pressure (350 kPa), followed by a decrease from 50 to 180 mmHg. At 4 MHz and 450–500-kPa acoustic pressures, Li *et al.* [17] found a decrease in the subharmonic signal with hydrostatic pressure, as would be expected from previous work with other ultrasound contrast agents [10]. Of specific relevance to the investigation of SonoVue as a potential pressure sensor is the work by Frinking *et al.* [11] that examined SonoVue-like microbubbles from Bracco Research—they found an increase in the subharmonic signal of SonoVue-like microbubbles to hydrostatic pressure at 4 MHz and 50-kPa acoustic pressure and no change in the subharmonic signal at the same transmit frequency (4 MHz) and 200 kPa acoustic pressure, but a decrease at 400-kPa acoustic pressure. The conflicting data in the literature mean that the subharmonic response of SonoVue to hydrostatic pressure has not been established and is still unclear. Building upon the extensive work led by Forsberg and colleagues toward developing Sonazoid as a pressure sensor, it is probable that an experimental protocol similar to that being used with Sonazoid may help clarify the subharmonic response of SonoVue to hydrostatic pressure.

The aim of this study was to investigate the subharmonic response of the ultrasound contrast agent SonoVue at physiological pressures using a static pressure phantom. We hypothesized that the subharmonic signal of SonoVue would exhibit: 1) a growth phase with increasing acoustic pressures and 2) a negative linear relationship with hydrostatic pressure at an acoustic pressure within this growth phase. The Rayleigh–Plesset equation for single bubble oscillations, combined with an effective bubble surface tension [19], and additional hysteresis experiments were used to provide insight into the mechanisms underlying the empirically observed subharmonic-pressure response. Part of this work, limited to the data at a transmit frequency of 5 MHz, was first presented at the 2017 IEEE International Ultrasonics Symposium [20].

## II. Materials and Methods

### A. Static Pressure Chamber

A phantom capable of maintaining 0–200-mmHg static hydrostatic pressures was developed using a cell culture cassette with Luer connections (CLINIcell 25, 175-m membrane, 10-mL volume, 6.8 cm $\times3.9$ cm $\times3.7$ mm, Mabio International, Tourcoing, France) and submerged in a water bath (see Fig. 1). Luer connections ensured that the CLINIcell chamber was airtight to maintain stable hydrostatic pressures. A similar cell culture cassette albeit with a thinner membrane (50-m membrane) has been recently demonstrated as a viable chamber for microbubble studies [21]. The ultrasound transducer was positioned at a 45° angle relative to the cell culture cassette [22]. This enabled a clear region of interest with minimal backscatter from the cassette windows and concomitantly increased the effective depth of the pressure chamber on the ultrasound image to 5.2 mm. A 1.5-mm magnetic stirrer was inserted into the cassette to maintain a homogenous concentration of microbubbles within the pressure chamber.

High-pressure polyvinyl chloride (PVC) tubing was secured to the cassette via the two Luer ports (900 PSI, Cole-Parmer, Cambridgeshire, U.K.) and led out of the water bath to entry and exit Luer stopcocks for administering microbubble solution. Prior to the exit stopcock, a pressure sensor (PRESS-S-000 sensor, PendoTech, Princeton, NJ, USA) was connected and positioned outside the water bath at the same height as the middle of the submerged cassette. The pressure sensor was connected to a digital pressure meter (INFCS-112B meter, Newport Electronics, Inc., Santa Ana, CA, USA) calibrated at 0 mmHg (ambient pressure) and 147 mmHg using a water column (2-m water column).

### B. Attenuation and Insertion Loss

A 12-mm layer of open-cell melamine foam (Basotect, BASF, Ludwigshafen, Germany) was positioned in front of the cassette to create an attenuating layer between the ultrasound transducer and the microbubbles. To measure the attenuation resulting from this foam and the insertion loss through one window of the cell culture cassette, a pair of broadband transducers (Panametrics V311, 12.7-mm diameter, 59-mm focal length, 10-MHz center frequency; Panametrics V310, 6.35-mm diameter, 5-MHz center frequency; Olympus NDT, Waltham, MA, USA) were used to acquire the through-transmission spectrum using a broadband substitution technique [23]. Measurements were conducted in an 8-L acrylic tank ($45\times 12\times15$ cm) filled with distilled water. An ultrasound pulser–receiver (DPR300, JSR Ultrasonics, Pittsford, NY, USA) was used to generate the excitation pulse and amplify the received signal (20–50-dB gain). Received waveforms were averaged (typically 64 traces/acquisition), digitized (LT264, LeCroy, Chestnut Ridge, NY, USA), and transferred to a computer for analysis using MATLAB (The MathWorks, Inc., Natick, MA, USA). Attenuation through the open-cell melamine foam and insertion loss through one window of the CLINIcell were used to calculate the total acoustic signal loss and to estimate the incident acoustic pressure within the cassette chamber (acoustic pressure in a water bath $\times \,\,10^{-\mathrm {total\,\,signal\,\,loss}/20}$). All tables and figures show peak-negative acoustic pressure corrected for signal loss, unless stated otherwise.

Layers of acoustically absorbent open-cell foam were additionally positioned behind the cassette to reduce artifacts created due to reflections and scattering beyond the cassette chamber.

### C. Experiments With SonoVue at 0–200-mmHg Hydrostatic Pressures

SonoVue was reconstituted according to the manufacturer’s instructions and diluted in gas-equilibrated water to yield the typical concentration used in the clinic (0.4-$\mu \text{L}$/mL water). With the exit port open and the magnetic stirrer spinning in the cassette chamber, approximately 25 mL of diluted microbubble solution was added to the static pressure phantom ($\approx 0.5$ mL/s). The exit port was then closed and the hydrostatic pressure was increased by adding more microbubble solution.

Radio frequency data were recorded across the bandwidths of a linear and a phased array ultrasound transducer (bandwidth 3–7 MHz, LA332E Marzo 2014 and bandwidth 1.2–2.1 MHz, PA230, respectively; Esaote, Genoa, Italy) on the ULtrasound Advanced Open Platform (ULA-OP; MSD Lab, University of Florence, Florence, Italy). These transducers were used in this study to encompass vascular and cardiac imaging.

Pulse-inversion sequences and long transmit pulses were used to enhance the nonlinear microbubble signal [18], [24]. A longer transmit pulse has been found to enhance the subharmonic signal-to-noise ratio and reduce the transient effect of pulse length due to the growth and decay at the beginning and end of each ultrasound pulse on the subharmonic signal [18], [24]. Therefore, pulse length was chosen to maximize the number of cycles per pulse while not exceeding the 5.2-mm effective chamber depth (with a 45° angle of insonation). Sixteen-cycle pulses were used at transmit frequencies 5–7 MHz, 12-cycle pulses at 4 MHz, 10-cycle pulses at 3 MHz, 7-cycle pulses at 2.1 MHz, and 5-cycle pulses at 1.7 MHz. The pulse length at transmit frequency 5 MHz with 16 cycles was 4.14 mm (standard deviation 0.02 mm, $n=3$, 82% maximum scanner acoustic output). The maximum mechanical index on the linear transducer was 0.52 at transmit frequency 6 MHz with 16-cycle pulses, and the maximum mechanical index on the phased array transducer was 0.41 at transmit frequency 2.1 MHz with 7-cycle pulses, as measured with a 0.5-mm hydrophone (SN1832, Precision Acoustics Ltd., Dorchester, U.K.) in a water bath.

Data were first recorded at ambient hydrostatic pressure (0 mmHg) from 3.5% to 100% maximum scanner acoustic output ($n=40$, equally spaced on a logarithmic scale; 9 min per data set of incremental acoustic outputs) [13] —to determine the acoustic pressure range that elicited the growth phase response of SonoVue. Subsequently, data were recorded across the scanner acoustic output levels corresponding to the growth phase ($n=20$; 4 min per data set).

Experiments were performed from 200- to 0-mmHg hydrostatic pressures in 25-mmHg decrements and then repeated. The microbubble solution in the phantom was replenished after each set of acoustic output levels. Experiments at individual hydrostatic pressure levels were further repeated if no crossover was observed between the first two sets (i.e., if one set of data points was consistently higher than the other set within the growth phase). The erroneous data set was determined based on its large variation from the other two runs that had intersecting growth phases, attributed to human error in replenishing the microbubble solution or noise [25], and discarded. Across the transmit frequencies investigated, additional runs were recorded at 2–6 pressure levels (out of 9) to obtain intersecting growth phases (i.e., 1.7 MHz: 0, 50, 75, 100, 125, and 150 mmHg; 2.1 MHz: 175 and 200 mmHg; 3 MHz: 0, 25, 100, 125, 150, and 175 mmHg; 4 MHz: 0, 25, 150, 175, and 200 mmHg; 5 MHz: 0, 25, 100, and 175 mmHg; 6 MHz: 0, 25, 50, 150, 175, and 200 mmHg; and 7 MHz: 0, 175, and 200 mmHg). Noisy subharmonic data are common and can be mitigated *in vivo* by applying a median filter on a larger number of frames [26]. All experiments were performed at room temperature ($\approx 21~^\circ \text{C}$) and were completed within 7 h of microbubble reconstitution. Crossover of the data between repeats indicated that the subharmonic signal of SonoVue was stable across this period.

### D. Extracting and Analyzing the Subharmonic Signal

The mean signal amplitude over a 40% bandwidth around the nominal subharmonic frequency (i.e., transmit frequency $f_{0}/2$) was extracted off-line using MATLAB [26]. A zero-phase digital filter was applied with a finite impulse response (FIR) bandpass filter to isolate the signal over the subharmonic bandwidth. In experiments with the linear transducer, a 15.5 $\times $ 3 mm region of interest was defined based on the B-mode image at the center frequency of 5 MHz with 16-cycle pulses (see Fig. 2). In experiments with the phased array transducer, a sector region of interest with 3-mm depth was defined from the B-mode image at a transmit frequency of 2.1 MHz with 7-cycle pulses. The average subharmonic amplitude was calculated as the mean amplitude across three frames of the region of interest (i.e., 3 frames $\times $ 64 lines/frame).

Linear regressions between subharmonic amplitude and hydrostatic pressure were performed at each acoustic output level to identify the maximum sensitivity of SonoVue to changes in hydrostatic pressure. Mean error was calculated as the mean absolute difference between the data and the regression line.

### E. Using the Rayleigh–Plesset Equation to Investigate the Subharmonic-Pressure Relationship of SonoVue

To gain first insights into the mechanisms underlying the subharmonic-pressure relationship of SonoVue, we used a classical model for single-bubble oscillations described by the Rayleigh–Plesset equation and assumed an adiabatic gas response. The Peclet number for a 2-$\mu \text{m}$ air bubble at 1 MHz is 120, and therefore, the adiabatic assumption (Pe $\gg $ 1) is verified [27]. This provided the amplitude of bubble oscillation as a function of hydrostatic pressure when the bubble is excited with a pure sinusoidal wave of known frequency $f$ and a fixed peak-to-peak acoustic pressure of 150 kPa. The equilibrium bubble radius $R_{0}$ at a given pressure $p$ was obtained by assuming that the amount of gas inside the bubble remains constant

\begin{equation*} R^{3}_{0} \left({p + \frac {2\sigma }{R_{0}} }\right) = C_{\mathrm {ref}} \tag{1}\end{equation*}

where $C_{\mathrm {ref}}$ is a constant obtained from the reference radius $R_{\mathrm {ref}}$ measured at the reference pressure. $R_{\mathrm {ref}} = 2\,\,\mu \text{m}$, using the typical modal radius of SonoVue at reference pressure [28].

The amplitude of SonoVue bubble oscillations is additionally affected by buckling of its phospholipid monolayer [19], [28]. As hydrostatic pressure increases, bubble radius decreases and buckling occurs below a critical radius, which is dependent on the number of phospholipid molecules surrounding the bubble. Effective surface tension $\sigma $ of the lipid monolayer ranges from 0.07 N/m in the elastic state for air/water systems to 0 N/m in the buckled state (1). A parametric study was performed to investigate the influence of surface tension on the amplitude of bubble oscillations.

We then used the predicted oscillation amplitude from the single-bubble model to estimate reflected sound as a function of hydrostatic pressure. In the weakly nonlinear regime, it is reasonable to assume that the intensity of reflected sound is proportional to the intensity of nonlinearity [29] and inversely proportional to the void fraction [30]. These parameters are represented in (2) as bubble oscillation amplitude and equilibrium pressure ($p_{0}$), respectively. If we adjust the model using the signal intensity at the reference pressure for air/water bubbles, the predicted amplitude $A$ in dB across 0–200-mmHg equilibrium pressures $p_{0}$ is

\begin{equation*} A = 27 + 20 \log \left ({\frac {\Delta R_{0}}{\Delta R_{\mathrm {ref}}} \frac {p_{\mathrm {ref}}}{p_{0}}}\right). \tag{2}\end{equation*}

### F. Hysteresis Experiments to Investigate the Irreversible Impact of Hydrostatic Pressure on SonoVue

SonoVue was added to the static pressure phantom following the experimental protocol in Section II-C. A transmit frequency of 5 MHz with 16-cycle pulses was used for this set of experiments. Similar to the experiments in Section II-C, radio frequency data were first recorded at ambient pressure (0 mmHg) to determine the range of scanner acoustic output levels corresponding to the growth phase ($n=40$).

To investigate the irreversible impact of hydrostatic pressure on SonoVue, the microbubble solution in the phantom was first maintained at 200–0 mmHg without insonation. The pressure chamber was then returned to 0 mmHg, and data were recorded at the scanner acoustic output levels corresponding to the growth phase ($n=20$). The SonoVue solution in the phantom was replenished after each set of acoustic output levels. Experiments were performed in 25-mmHg decrements. Three sets of data were recorded for each hydrostatic pressure level, and experiments were completed within 4.5 h of microbubble reconstitution.

## III. Results

### A. Acoustic Signal Loss Before Reaching the Microbubbles

At the 45° angle of insonation used in this study, total attenuation through the foam and the single cassette window ranged from 4.6 to 16.2 dB across 1.7–7.0-MHz transmit frequencies (see Tables I and II). The insertion loss across 0°–50° angles of insonation are reported in the Appendix.

**TABLE I:** Attenuation Through Open-Cell Melamine Foam (Basotect, BASF, Ludwigshafen, Germany) Across 1–7-MHz Transmit Frequencies. Values are Mean (Standard Deviation) of Two Measurements

Frequency (MHz)	Attenuation (dB/cm)
1.0	0.89 (0.44)
2.0	1.00 (0.49)
3.0	1.12 (0.61)
4.0	1.35 (0.66)
5.0	1.75 (0.60)
6.0	2.15 (0.55)
7.0	2.51 (0.50)

**TABLE II:** Insertion Loss Through One Window of the CLINIcell Chamber at a 45° Angle of Insonation, Across 1–7-MHz Transmit Frequencies. Values are Mean (Standard Deviation) of Four Measurements

Frequency (MHz)	Insertion loss (dB)
1.0	0.92 (0.05)
1.5	2.04 (0.06)
2.0	4.36 (0.08)
2.5	10.45 (0.28)
3.0	14.30 (0.39)
3.5	4.34 (0.11)
4.0	2.63 (0.05)
4.5	2.72 (0.09)
5.0	3.22 (0.17)
5.5	3.64 (0.14)
6.0	4.25 (0.03)
6.5	7.23 (0.16)
7.0	10.50 (0.57)

### B. Occurrence, Growth, and Saturation Phases of SonoVue With Increasing Acoustic Pressure

SonoVue generated subharmonic signals that followed the characteristic occurrence, growth, and saturation phases previously observed in microbubble ultrasound contrast agents [13] (representative example in Fig. 3). The acoustic pressures corresponding to these phases varied across transmit frequencies, with the growth phase occurring between 50- and 250-kPa peak-negative pressure.

### C. Ascending and Descending Phases of the Subharmonic Amplitude of SonoVue With Hydrostatic Pressure

**Fig. 4.**
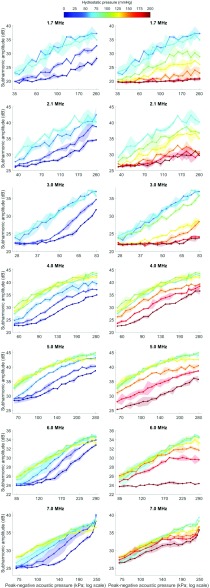
Mean subharmonic amplitude of SonoVue at 0–200-mmHg hydrostatic pressures, across transmit frequencies 1.7 and 2.1 MHz with the phased array transducer and 3.0–7.0 MHz with the linear transducer (${n} \,\,={2}$). Left column: ascending phase of the subharmonic-pressure relationship. Right column: descending phase. Data at the plateau phase are repeated in both columns for reference. Translucent shading indicates 1 standard deviation around the mean. The horizontal axis differs between rows for clarity of the individual plots.

From 0- to 50-mmHg hydrostatic pressure, the subharmonic signal of SonoVue increased for all transmit frequencies investigated in this study (1.7–7.0 MHz; see Fig. 4). Between 50- and 75-mmHg hydrostatic pressure, the subharmonic signal further increased for most of the transmit frequencies except at 1.7 and 3.0 MHz, which corresponded to the lowest transmit frequencies investigated for both the linear and phased array transducers. For these transmit frequencies, the subharmonic signal plateaued between 50 and 75 mmHg.

**Fig. 5.**
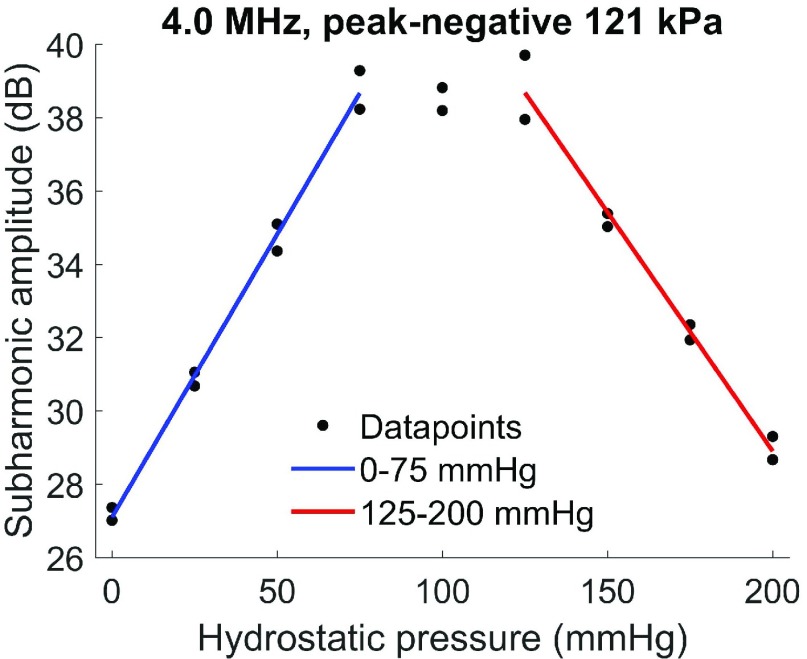
Subharmonic amplitude of SonoVue from 0- to 200-mmHg hydrostatic pressure at a transmit frequency of 4 MHz and 121-kPa peak-negative acoustic pressure (${n} ={2}$). Sensitivity of the subharmonic signal to 0–75-mmHg hydrostatic pressure was 0.15 dB/mmHg (${r} ^{{2}}={0.99}$; ${p} < {0.001}$).

Above 75 mmHg, the subharmonic signal of SonoVue decreased as hydrostatic pressure increased for transmit frequencies 1.7–3.0 MHz. For transmit frequencies 4.0–7.0 MHz, however, the subharmonic signal plateaued between 75- and 125-mmHg hydrostatic pressure and only decreased as hydrostatic pressures were increased above 125 mmHg. The maximum sensitivity of the subharmonic-pressure relationship for each transmit configuration was identified for 0–75-mmHg and 125–200-mmHg hydrostatic pressures separately and summarized in Tables III and IV and Fig. 5.

**TABLE III:** Maximum Sensitivity of the Subharmonic Amplitude of SonoVue to 0-75-mmHg Hydrostatic Pressure Across 1.7–7.0-MHz Transmit Frequencies

Linear regression results	1.7 MHz	2.1 MHz	3.0 MHz	4.0 MHz	5.0 MHz	6.0 MHz	7.0 MHz
Maximum sensitivity (dB/mmHg)	0.16	0.15	0.13	0.15	0.13	0.07	0.09
Mean error (mmHg)	13.9	4.4	7.6	2.0	1.4	5.6	9.6
Peak-negative pressure (kPa)	126	155	53	121	141	153	140
Mechanical index	0.16	0.24	0.20	0.11	0.13	0.16	0.29
Adjusted $r^{2}$	0.677	0.957	0.894	0.992	0.995	0.938	0.820
$p$	0.007	<0.001	<0.001	<0.001	<0.001	<0.001	0.001

**TABLE IV:** Maximum Sensitivity of the Subharmonic Amplitude of SonoVue to 125–200-mmHg Hydrostatic Pressure Across 1.7–7.0-MHz Transmit Frequencies

Linear regression results	1.7 MHz	2.1 MHz	3.0 MHz	4.0 MHz	5.0 MHz	6.0 MHz	7.0 MHz
Maximum sensitivity (dB/mmHg)	−0.09	−0.09	−0.07	−0.14	−0.16	−0.13	−0.05
Mean error (mmHg)	9.2	7.1	10.9	2.6	3.6	6.9	8.5
Peak-negative pressure (kPa)	233	155	78	101	131	273	191
Mechanical index	0.30	0.24	0.29	0.09	0.12	0.28	0.40
Adjusted $r^{2}$	0.871	0.911	0.811	0.986	0.974	0.910	0.864
$p$	<0.001	<0.001	0.001	<0.001	<0.001	<0.001	<0.001

### D. Effects of Hydrostatic Pressure and Surface Tension Using the Rayleigh–Plesset Equation

**Fig. 6.**
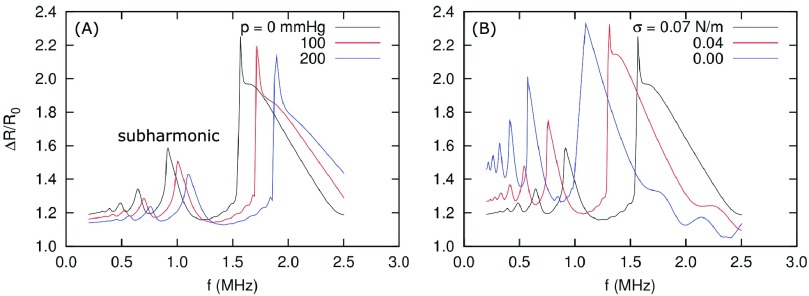
Amplitude of bubble oscillation using the Rayleigh–Plesset equation with 150-kPa peak-to-peak acoustic pressure. (a) Simulation results at 0-, 100-, and 200-mmHg hydrostatic pressures. (b) Simulation results at and below the surface tension corresponding to air/water systems (0–0.07 N/m).

**Fig. 7.**
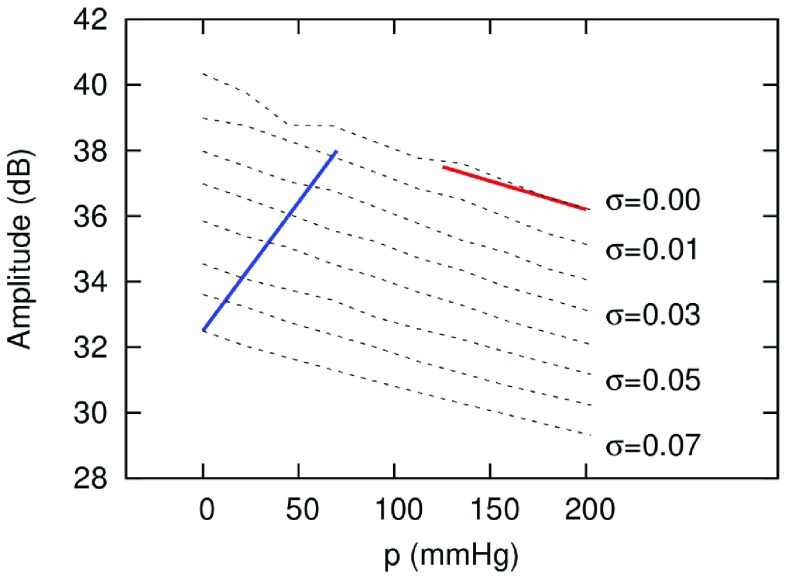
Predicted subharmonic amplitude across 0–200-mmHg hydrostatic pressures and 0.00–0.07-N/m bubble surface tension, derived using the Rayleigh–Plesset equation with a bubble radius of 2 $\mu \text{m}$. The blue line shows a possible increase of the subharmonic signal with decreasing surface tension; the red line marks the decrease in the subharmonic signal with increasing hydrostatic pressure at zero surface tension.

At a constant surface tension ($\sigma =0.07$ N/m), the simulations revealed a decrease in the subharmonic amplitude with an increasing hydrostatic pressure [see Fig. 6(a)]. In contrast, at a constant hydrostatic pressure ($p =0$ mmHg), the subharmonic amplitude increased with a decreasing surface tension [see Fig. 6(b)]. The predicted subharmonic amplitude in dB as a function of hydrostatic pressure and surface tension is shown in Fig. 7.

### E. Effects of Prior Exposure to 0–200-mmHg Hydrostatic Pressures on the Subharmonic Signal of SonoVue

**Fig. 8.**
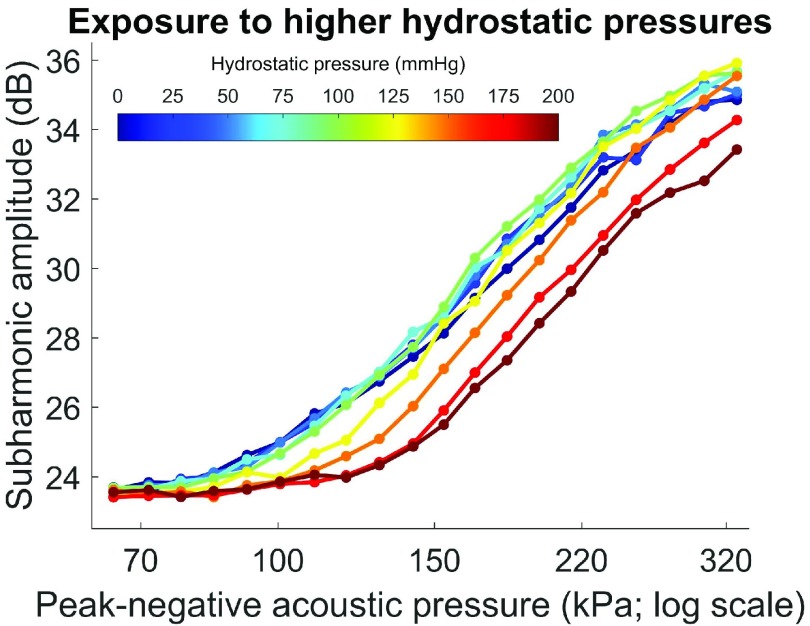
Mean subharmonic amplitude of SonoVue at ambient hydrostatic pressure (0 mmHg), after 1 min of exposure to higher pressures (${n} \,\,={3}$; standard deviations omitted for clarity). Data recorded at a transmit frequency of 5 MHz with 16-cycle pulses. Horizontal axis: water bath acoustic pressures corrected for 6.19-dB signal loss through the melamine foam and the cassette window.

The subharmonic amplitude of SonoVue was lower after exposure to 150–200-mmHg hydrostatic pressures, but not following exposure to 0–125 mmHg (see Fig. 8). The greatest decrease in the subharmonic amplitude between exposures to 125- and 200-mmHg hydrostatic pressures was 3.2 dB, which occurred at a 181-kPa peak-negative acoustic pressure (26.6% maximum scanner acoustic output).

## IV. Discussion

In this study, we investigated the subharmonic response of the microbubble ultrasound contrast agent SonoVue at 0–200-mmHg hydrostatic pressures using a static pressure phantom. We developed a new phantom from commercially available components, an acquisition protocol to record radio frequency data at incremental acoustic output levels and a signal processing toolbox to extract the subharmonic amplitude from the received signal. The subharmonic signal of SonoVue increased from 0- to 50-mmHg hydrostatic pressure across 1.7–7.0-MHz transmit frequencies and decreased from 125 to 200 mmHg. Decreasing surface tension may explain the increase in the subharmonic signal from 0- to 50-mmHg, while shell buckling and bubble destruction likely contribute to the decrease in the subharmonic signal from 125 to 200 mmHg.

### A. Optimal Transmit Frequency to Use the Subharmonic Signal of SonoVue to Estimate Hydrostatic Pressure

Across the transmit frequencies investigated, 4.0 MHz elicited the best subharmonic sensitivity for assessing hydrostatic pressures up to 75 mmHg (0.15 dB/mmHg). Comparable sensitivities to hydrostatic pressure were found for transmit frequencies 1.7–3.0 MHz, but the decrease in the subharmonic signal from 75 to 125 mmHg would result in nonunique values that may be incorrectly interpreted when used to estimate pressures *in vivo*. This is not a concern at a transmit frequency of 4.0 MHz because the subharmonic signal plateaus from 75 to 125 mmHg. In addition, the transmit frequencies 1.7–5.0 MHz involved extracting the subharmonic signal outside the bandwidth of the transducers used in this study. Future use of a transducer with a bandwidth that includes both the subharmonic and transmit frequencies may thus further increase upon the sensitivities found in this study. Nonetheless, the ascending and descending pattern of the subharmonic-pressure relationship is a unique characteristic of SonoVue bubble behavior and independent of the choice of the transducer.

### B. Subharmonic-Pressure Response of SonoVue Differs From Other Microbubble Ultrasound Contrast Agents

The observed increase in the subharmonic signal of SonoVue from 0- to 75-mmHg hydrostatic pressure was in stark contrast to our hypothesis predicting a linear decrease from 0 to 200 mmHg. Our hypothesis was based upon previous work that found this linear decrease of the subharmonic signal across multiple ultrasound contrast agents—Sonazoid, Optison, Levovist, and Definity—but SonoVue was not investigated in that study [10]. In agreement with our findings, Li *et al.* [17] have recently reported an increase in the subharmonic amplitude of SonoVue from 0- to 50-mmHg hydrostatic pressure, followed by a decrease from 50 to 180 mmHg, at a transmit frequency of 4 MHz with a peak-negative acoustic pressure of 350 kPa. At 450-kPa peak-negative acoustic pressure and even higher mechanical indices (≥0.225), however, Li *et al.* [17] observed a decrease in the subharmonic amplitude of SonoVue with hydrostatic pressure. This may be due to microbubble destruction at higher mechanical indices and thus demonstrates the need for low mechanical index imaging in this new application of using SonoVue microbubbles to estimate hydrostatic pressure. A direct comparison of the subharmonic response of SonoVue with other microbubble ultrasound contrast agents will help verify this different behavior of SonoVue [31].

### C. Decreasing Surface Tension May Underpin the Ascending Phase of the SonoVue Subharmonic-Pressure Relationship

Our simulations using the Rayleigh–Plesset equation revealed an increase in the subharmonic signal with decreasing bubble surface tension and a decrease in the subharmonic signal with increasing hydrostatic pressure. These competing effects of hydrostatic pressure and surface tension likely underpin the resulting net subharmonic signal observed experimentally from SonoVue microbubbles. Based on our simulation results, we speculate that the observed increase in the subharmonic signal of SonoVue from 0- to 75-mmHg pressure was driven by a decreasing microbubble surface tension (see Fig. 7). With decreasing surface tension, the oscillations of microbubbles shift toward more compression than expansion [11]. In addition to SonoVue microbubbles, which consist of sulfur hexafluoride gas (SF_6_) in a phospholipid shell, this “compression-only” behavior has been implicated in the increased subharmonic signal of experimental perfluorobutane (C_4_F_10_) phospholipid-shell microbubbles with hydrostatic pressure [11].

### D. Microbubble Buckling and Destruction Contribute to the Descending Phase of the SonoVue Subharmonic-Pressure Relationship

Above 125-mmHg hydrostatic pressure, we speculate that most of the SonoVue microbubbles are in a buckled state (i.e., zero surface tension), and thus, the subharmonic signal of SonoVue decreases with increasing hydrostatic pressure (see Fig. 7) [19]. However, the extent of the decrease of the subharmonic signal observed experimentally was greater than that predicted by our simulations using the Rayleigh–Plesset equation. This discrepancy may be explained by irreversible bubble destruction at 150–200-mmHg hydrostatic pressures, which would violate the assumption that changes in void fraction solely reflect changes in bubble volume ($v$)—and not fewer bubbles per unit volume [see (2); $({v_{\mathrm {gas}}}/({v_{\mathrm {gas\,\,reference}}}) = ({p_{\mathrm {ref}}})/{p_{0}}$]. Indeed, the lower subharmonic signal observed in the hysteresis experiments following exposure to 150–200-mmHg for 1 min, but not following exposure to 0–125 mmHg, supports the inference of microbubble destruction above 125 mmHg. Destruction of SonoVue microbubbles upon exposure to higher hydrostatic pressures may include lipid shedding from the bubble shell and static diffusion of SF_6_ gas out of the bubble core, followed by inertial cavitation and fragmentation of the bubble when the pressure is released [32]–[34].

Taken together, the descending phase of the subharmonic-pressure relationship of SonoVue from 125 to 200 mmHg is likely underpinned by both bubble buckling and bubble destruction. Our findings additionally reiterate the importance of empirical data in investigations of microbubble ultrasound contrast agents, as existing mathematical models do not yet fully characterize complex microbubble mechanics [12], [35].

### E. Clinical Implications

The subharmonic-pressure relationship of SonoVue may be used to estimate pressures *in vivo*. While systolic and diastolic brachial artery pressures are measured routinely using a sphygmomanometer, blood pressures in the heart and aorta are currently assessed invasively using a pressure catheter. The linear increase in subharmonic amplitude from 0 to 75 mmHg may be applied to estimate left ventricular diastolic pressures (4–12 mmHg), which are critical for the assessment of diastolic performance [8], [9]. This pressure range is also appropriate for the assessment of right ventricular pressure (2–30 mmHg) and left (4–12 mmHg) and right atrial pressures (2–6 mmHg) across the cardiac cycle. On the other hand, the linear decrease in the subharmonic signal above 75 mmHg may be applied to assess aortic pressures across the cardiac cycle (80–120 mmHg). For clinical applications, an estimate of pressure within 5 mmHg of reference pressure is ideal [36] and appears to be most likely achievable with SonoVue at transmit frequencies of 2.1, 4.0, and 5.0 MHz.

One possible obstacle in translating our ultrasound transmit and receive configurations to image the heart and great vessels could be a greater attenuation through the body than through the static pressure phantom in this study. However, the attenuation through 1 cm of septal or lateral myocardium at a transmit frequency of 2.1 MHz is less than 3 dB [37], [38], which is less than the measured 4.6–16.2-dB signal loss in our phantom. It is thus likely that the transmit and receive configurations developed in this study can be successfully translated to *in vivo* imaging. In addition, the transducers used in this study are already routinely used for cardiac and vascular imaging.

In addition to SonoVue, other microbubble ultrasound contrast agents, such as Sonazoid and Definity, have been investigated as potential pressure sensors. These have shown potential as noninvasive pressure sensors in the right ventricle [5], for diagnosing portal hypertension [39], and for estimating tumor interstitial fluid pressure in breast cancer [40]. One disadvantage of using SonoVue, compared with Sonazoid or Definity, is its nonunique subharmonic amplitudes from 0- to 200-mmHg hydrostatic pressures. However, an advantage of SonoVue is its greater sensitivity to changes in pressure, as sensitivities of up to ±0.16 dB/mmHg were found in this study. An earlier study by Halldorsdottir *et al.* [10] comparing different microbubble ultrasound agents found lower sensitivities for Sonazoid (−0.08 dB/mmHg), Definity (−0.07 dB/mmHg), and Optison (−0.06 dB/mmHg), but did not investigate SonoVue. Optimization of the ultrasound pulse shape subsequently improved the sensitivity of Sonazoid to −0.17 dB/mmHg for 0–40-mmHg hydrostatic pressures, but the maximum pressure range was limited by the decrease of the subharmonic signal below the noise floor [41]. Despite extensive research on Sonazoid, one of its limitations is that it is currently not approved for use in Europe [15]. The investigation of SonoVue, which is widely used in Europe, may help accelerate this exciting new technology from the lab to the clinic in Europe. Altogether, these characteristics need to be considered and balanced to ultimately choose the best possible microbubble ultrasound contrast agent for the target application in the human body.

### F. Limitations and Future Work

This work included the development of a new static pressure phantom, suitable for microbubble experiments [20]. As the probe and the cell culture cassette were positioned manually without using a rotation mount, the angle of incidence may not have been precisely 45°. Manual measurement with a protractor, however, allowed us to estimate the angle of incidence and with this, calculate a best-estimate of the signal loss prior to the pressure chamber. Due to resource limitations, our pressure meter was calibrated with a simple 2-m water column and only reached 147-mmHg hydrostatic pressure. To achieve a higher accuracy of pressures above 147 mmHg on the pressure meter, a pneumatic calibrator may be used in the future. In addition, only two sets of data were acquired at each hydrostatic pressure level and more sets would have been ideal. However, this was the result of a balance between minimizing the duration of experiments from the point of microbubble reconstitution (to minimize differences in microbubble properties over time) and maximizing the number of hydrostatic pressure levels ($n=9$) and acoustic outputs investigated ($n=20$). A visual inspection of the first two sets of data to determine any crossover of the data points was used instead to exclude erroneous data sets and to ensure the best estimate of the subharmonic amplitude of SonoVue in this study.

To provide first insights into the mechanisms underlying the subharmonic-pressure relationship of SonoVue, we used a model of a pure gas bubble with an effective surface tension [19], coupled with a simple representation of the bubble cloud response. This provided qualitative insight into our experimental findings but does not fully represent the complex interaction between bubble clusters and acoustic waves [35]. Further work using more comprehensive and elaborate models, such as the subgrid model for bubbly cavitating flows proposed by Fuster and Colonius [42], will enable a better representation of the bubble cloud beyond commonly used single-bubble models [12].

Building upon the ultrasound acquisition protocol and the signal processing toolbox developed in this study, future work will include shortening the protocol to be able to complete this test within 20 min in the clinic (the experimental protocol in this study investigating 0–200-mmHg hydrostatic pressures at one transmit frequency took up to 4.5 h to complete). This will likely include quicker identification of the optimal acoustic output and subsequent data collection across time at this single acoustic output [26], [43]. As differences in attenuation, blood viscosity [44], and temperature [45] likely affect the subharmonic signal of SonoVue, we envision that an incremental acoustic output scan will be necessary for each patient acquisition to identify an individualized optimal acoustic output, similar to previous work on Sonazoid [26] and Definity [5]. Studies conducted *in vitro* have found that an increase in temperature from room temperature to body temperature decreases microbubble stability [45], while an increase in viscosity from water to blood has the opposite effect of increasing microbubble stability [44]. Future experiments at body temperature and in a blood-mimicking fluid medium (instead of at room temperature and in water in this study) will therefore enable optimization of this technique prior to *in vivo* testing. In addition, amplitude modulation and pulse shaping may be investigated as methods to further enhance the subharmonic signal of SonoVue [41], [46].

## V. Conclusion

The subharmonic signal of SonoVue first increased with hydrostatic pressure across all experimental conditions (0–50 mmHg) and then decreased (125–200 mmHg). The increase in the subharmonic signal of SonoVue may be driven by a decreasing bubble surface tension, while the decrease may be attributed to both shell buckling and bubble destruction. Our results report the largest sensitivity to date across 0–200 mmHg (±0.16 dB/mmHg), opening promising translational perspectives for a less invasive method to assess diastolic filling pressures (compared with inserting a catheter into the heart).

## Appendix Characterization of the Insertion Loss Through One Window of the CLINIcell Chamber

Table V shows the insertion loss through one window of the CLINIcell chamber at 0°–50° angles of insonation, across 1–7-MHz transmit frequencies. Future ultrasound studies using the CLINIcell cell culture cassette with 175-$\mu \text{m}$ membrane can use this detailed characterization to calculate the acoustic signal loss in their experimental setups.

**TABLE V:** Characterization of the Insertion Loss (dB) Through One Window of the CLINIcell Chamber. Values are Mean (Standard Deviation) of Four Measurements

Transmit frequency (MHz)	Angle (deg)										
	0	5	10	15	20	25	30	35	40	45	50
1.0	0.38 (0.08)	0.35 (0.09)	0.38 (0.08)	0.34 (0.10)	0.33 (0.01)	0.33 (0.11)	0.24 (0.03)	0.17 (0.06)	0.43 (0.05)	0.92 (0.05)	1.91 (0.20)
1.5	0.68 (0.02)	0.69 (0.02)	0.69 (0.04)	0.75 (0.05)	0.78 (0.03)	0.86 (0.03)	0.96 (0.02)	1.01 (0.01)	1.33 (0.04)	2.04 (0.06)	4.28 (0.12)
2.0	1.11 (0.02)	1.13 (0.01)	1.20 (0.00)	1.29 (0.02)	1.43 (0.03)	1.61 (0.03)	1.84 (0.03)	2.12 (0.02)	2.79 (0.04)	4.36 (0.08)	8.84 (0.17)
2.5	1.41 (0.03)	1.72 (0.02)	2.38 (0.03)	3.06 (0.04)	3.58 (0.01)	3.94 (0.04)	4.39 (0.05)	5.02 (0.06)	6.50 (0.04)	10.45 (0.28)	24.25 (0.74)
3.0	1.53 (0.02)	1.46 (0.02)	1.33 (0.04)	1.37 (0.05)	1.95 (0.04)	4.02 (0.07)	7.96 (0.10)	12.16 (0.17)	15.64 (0.17)	14.30 (0.39)	7.92 (0.29)
3.5	1.52 (0.02)	1.48 (0.03)	1.37 (0.02)	1.24 (0.03)	1.12 (0.02)	1.18 (0.05)	1.70 (0.07)	2.82 (0.04)	4.07 (0.03)	4.34 (0.11)	3.51 (0.16)
4.0	1.42 (0.02)	1.40 (0.03)	1.31 (0.02)	1.23 (0.03)	1.14 (0.00)	1.11 (0.01)	1.26 (0.02)	1.60 (0.04)	2.13 (0.08)	2.63 (0.05)	2.99 (0.02)
4.5	1.20 (0.02)	1.19 (0.02)	1.13 (0.01)	1.08 (0.02)	1.04 (0.00)	1.09 (0.00)	1.23 (0.01)	1.51 (0.05)	1.99 (0.08)	2.72 (0.09)	3.44 (0.05)
5.0	0.96 (0.01)	1.01 (0.02)	1.19 (0.02)	1.44 (0.02)	1.64 (0.01)	1.84 (0.01)	1.95 (0.01)	2.02 (0.08)	2.35 (0.16)	3.22 (0.17)	3.93 (0.07)
5.5	0.83 (0.01)	1.32 (0.06)	2.67 (0.09)	4.50 (0.11)	6.20 (0.10)	7.43 (0.07)	7.70 (0.02)	6.19 (0.14)	3.40 (0.10)	3.64 (0.14)	4.05 (0.02)
6.0	0.65 (0.01)	0.78 (0.05)	1.18 (0.07)	1.83 (0.10)	2.64 (0.13)	3.45 (0.11)	4.00 (0.09)	4.00 (0.07)	3.53 (0.13)	4.25 (0.03)	3.96 (0.07)
6.5	0.68 (0.01)	0.72 (0.04)	0.87 (0.06)	1.15 (0.09)	1.62 (0.11)	2.25 (0.11)	2.80 (0.10)	3.13 (0.13)	3.71 (0.23)	7.23 (0.16)	4.89 (0.34)
7.0	0.88 (0.01)	0.86 (0.05)	0.87 (0.05)	0.95 (0.08)	1.23 (0.13)	1.73 (0.15)	2.30 (0.12)	2.93 (0.20)	4.27 (0.38)	10.50 (0.57)	7.21 (0.75)
